# Supplementary material for: Sorting at embryonic boundaries requires high heterotypic interfacial tension
Source: Nat Commun. 2017 Jul 31;8:157. doi: 10.1038/s41467-017-00146-x (PMC5537356; doi:10.1038/s41467-017-00146-x)
Supplement: Supplementary file 2 — Supplementary Software 1 [file 41467_2017_146_MOESM2_ESM.zip › PottsModel/SrcPottsModel/doc/model/Constants.Manager.html]

Constants.Manager


---


|  |  |  |
| --- | --- | --- |
| **Overview** \| **Package** \| **Class** \| **Use** \| **Tree** \| **Deprecated** \| **Index** \| **Help** | |  |
| **PREV CLASS**   **NEXT CLASS** | **FRAMES**    **NO FRAMES**     **All Classes** |  |
| SUMMARY: NESTED \| FIELD \| CONSTR \| METHOD | DETAIL: FIELD \| CONSTR \| METHOD |  |


---


## model Class Constants.Manager

```
java.lang.Object
  model.Constants.Manager
```

**Direct Known Subclasses:**: Constants.GuiManager, Constants.LatticeManager, Constants.MetaManager, Constants.ParameterManager, Constants.PathManager

**Enclosing class:**: Constants

---

``` private abstract class Constants.Manager extends java.lang.Object ```

---

| **Field Summary** | |
| --- | --- |
| `java.util.Properties` | `prop` |


**Constructor Summary**

`Constants.Manager(java.util.Properties p)`


| **Method Summary** | |
| --- | --- |
| `(package private) abstract  void` | `processProperties()` |

**Methods inherited from class java.lang.Object**

`clone, equals, finalize, getClass, hashCode, notify, notifyAll, toString, wait, wait, wait`

**Field Detail**

### prop

```
public final java.util.Properties prop
```


**Constructor Detail**

### Constants.Manager

```
public Constants.Manager(java.util.Properties p)
```


**Method Detail**

### processProperties

```
abstract void processProperties()
```


---


|  |  |  |
| --- | --- | --- |
| **Overview** \| **Package** \| **Class** \| **Use** \| **Tree** \| **Deprecated** \| **Index** \| **Help** | |  |
| **PREV CLASS**   **NEXT CLASS** | **FRAMES**    **NO FRAMES**     **All Classes** |  |
| SUMMARY: NESTED \| FIELD \| CONSTR \| METHOD | DETAIL: FIELD \| CONSTR \| METHOD |  |


---
